# An automatic restoration framework based on GPU-accelerated collateral filtering in brain MR images

**DOI:** 10.1186/s12880-019-0305-9

**Published:** 2019-01-19

**Authors:** Herng-Hua Chang, Cheng-Yuan Li

**Affiliations:** Computational Biomedical Engineering Laboratory (CBEL), Department of Engineering Science and Ocean Engineering, National Taiwan University, No. 1 Sec. 4 Roosevelt Road, Daan, 10617 Taipei, Taiwan

**Keywords:** Collateral filter, GPU, Parallel computing, Image feature, Neural networks, MRI

## Abstract

**Background:**

Image restoration is one of the fundamental and essential tasks within image processing. In medical imaging, developing an effective algorithm that can automatically remove random noise in brain magnetic resonance (MR) images is challenging. The collateral filter has been shown a more powerful algorithm than many existing methods. However, the computation of the collateral filter is more time-consuming and the selection of the filter parameters is also laborious. This paper proposes an automatic noise removal system based on the accelerated collateral filter for brain MR images.

**Methods:**

To solve these problems, we first accelerated the collateral filter with parallel computing using the graphics processing unit (GPU) architecture. We adopted the compute unified device architecture (CUDA), an application programming interface for the GPU by NVIDIA, to hasten the computation. Subsequently, the optimal filter parameters were selected and the automation was achieved by artificial neural networks. Specifically, an artificial neural network system associated with image feature analysis was adopted to establish the automatic image restoration framework. The best feature combination was selected by the paired t-test and the sequential forward floating selection (SFFS) methods.

**Results:**

Experimental results indicated that not only did the proposed automatic image restoration algorithm perform dramatically faster than the traditional collateral filter, but it also effectively removed the noise in a wide variety of brain MR images. A speed up gain of 34 was attained to process an MR image, which completed within 0.1 s. Representative illustrations of brain tumor images demonstrated the capability of identifying lesion boundaries, which outperformed many existing methods.

**Conclusions:**

We believe that our accelerated and automated restoration framework is promising for achieving robust filtering in many brain MR image restoration applications.

**Electronic supplementary material:**

The online version of this article (10.1186/s12880-019-0305-9) contains supplementary material, which is available to authorized users.

## Background

Since its invention in the 1970s, magnetic resonance imaging (MRI) has been an important imaging technique for noninvasive diagnosis of the human body. The applications of MRI in the brain result in diversified images for further processes such as tissue classification, segmentation and registration [1–3]. However, the random noise in MRI scanners inevitably causes deterioration of brain MR images [4]. Consequently, noise removal has become a crucial issue in brain MR image post-processing [5]. The Gaussian filter has been extensively adopted in many image processing applications for its simplicity. However, anatomical boundaries are inevitably blurred by this lowpass filter. In addition to the Gaussian filter, one of the well-known techniques for noise removal has been the bilateral filter, which was originally proposed by Tomasi and Manduci [6]. It has been widely utilized in many medical image denoising applications for decades. The bilateral filter is an effective filtering algorithm that can both remove the random noise and preserve edges rather than blurring the lines in images. An iterative version of the bilateral filter for MR image restoration was recently introduced [7].

One remarkable technique of using statistics strategies is the linear minimum mean squared error (LMMSE) estimator [8], which computes the local mean, the local variance, and the local mean square value of the input image. Another famous method is the anisotropic diffusion filter (ADF) [9], in which pixel intensities are averaged from neighbors in a prescribed window, whose dimension and shape are measured at every location. An appropriate function of the image gradient is constructed in accordance with the diffusion coefficient to encourage filtering within a region of interest in preference not to filtering across the boundaries. It is noted that the quality of the denoised image is greatly relevant to the number of iterations. Subsequently, Ferrari [10] proposed a mathematical framework to automatically determine the best number of iterations of the ADF method by utilizing the maximization of some evaluation index. Nevertheless, it has been adopted in many medical image restoration applications [11].

In contrast to many methods that mainly reply on local pixels within a small neighbourhood, the non-local means (NLM) filter detects repeated structures in the global domain [12]. The similarity between pixels is region-based comparison in that pixels far from the kernel center are not penalized due to the distance to the center. Based on an enhanced sparse representation in the transform domain, the block-matching and 3D filtering (BM3D) strategy [13] extended the nonlocal filtering techniques. The enhancement of the sparsity is accomplished by aggregating similar 2D fragments of the image into 3D arrays. Subsequently, the Wiener filter is employed in the collaborative filtering procedure to remove noise.

Motivated by the bilateral filter, the collateral filter [14] was recently proposed that introduced a median filter and an entropy function to the filtering framework. The median filter compensated the Gaussian filter for random noise reduction. It has been shown that the collateral filter is more powerful than the Gaussian filtering, bilateral filtering, and ADF methods in many scenarios [14]. However, comparing to these two filters, the collateral filter is more complicated and time consuming. A contemporary technique called the tensor processing unit (TPU) has shown its excellent computation and energy efficiency over existing frameworks [15]. However, this domain specific architecture is exclusively deployed in Google data centers. One realistic approach to speed up the denoising procedure is through the parallel programming using the compute unified device architecture (CUDA) [16, 17] with the graphics processing unit (GPU).

A GPU is a specialized processor that accelerates graphic operations on personal computers, workstations, and mobile devices. The GPU has more processor cores than a traditional central processing unit (CPU) and each core can process data simultaneously. Therefore, the GPU is well suited to handle tasks that can be parallelized. For example, GPU-based acceleration has been shown effective in segmentation and classification applications [18, 19]. Additionally, CUDA is a programming platform developed by NVIDIA for general purpose computation on GPU (GPGPU) on NVIDIA GPUs [20]. Many of NVIDIA graphics cards support CUDA (e.g., GeForce, Quadro, and Tesla). With CUDA, programmers can manage memory on the CPU as well as the GPU from the host. Once memory spaces on both host and device sides are allocated and data are transferred, the program can launch kernels for execution on the device.

A kernel is a function that can be called from the host and executed on the device. Programmers can design the code in the kernel function for parallel computing. The kernel function defines the operations for a single thread and thousands of threads execute the function code synchronously. A thread is the basic execution unit for parallel computing. After the thread completes its task in the kernel function, the data are transferred back to the host.

Based on the GPU architecture, one pixel in an image can be handled by one thread and thousands of threads can run synchronously. Each thread can access data from different types of memories such as local, global and shared memory. While local memory belongs to one specific thread, global memory is shared by all threads. Indeed, local memory is part of global memory and both is slower than shared memory. Threads are organized in blocks and a grid is a group of blocks. Each block has its shared memory that can be accessed by its own threads. Although shared memory’s size is smaller than global memory, the access time of shared memory is faster like cache [21].

The objective of this paper is two-fold. First, to accelerate the computation of the collateral filter through the adoption of the CUDA strategy. Second, to automate the collateral filtering process by the aid of image texture features associated with artificial neural networks [22]. We will show that a fast and automatic denoising system based on CUDA-based collateral filtering provides robust and accurate restoration results over many existing denoising methods.

## Methods

### CUDA-based collateral filtering

As described earlier, the collateral filter is a more effective denoising method than the bilateral filter in that it contains three different Gaussian functions [14]. Let (*θ*_*x*_, *θ*_*y*_) be the pixel location under consideration in the filtering image and ${\Psi}_{\theta_x,{\theta}_y}$ be the neighborhood of (*θ*_*x*_, *θ*_*y*_):

1 $$ {\Psi}_{\theta_x,{\theta}_y}=\left\{\left({\mu}_x,{\mu}_y\right):\Big({\mu}_x,{\mu}_y\left)\in \right[{\theta}_x-N,{\theta}_x+N\left]\times \right[{\theta}_y-N,{\theta}_y+N\Big]\right\} $$

where *N* is the filter radius. The Gaussian functions for the spatial, radiometric and median-filtered components are respectively defined as

2 $$ {W}_{\theta_x,{\theta}_y}^S\left({\mu}_x,{\mu}_y\right)=\exp \left[-\frac{{\left|\left({\mu}_x,{\mu}_y\right)-\Big({\theta}_x,{\theta}_y\Big)\right|}^2}{2{\sigma}_S^2}\right] $$

and

3 $$ {W}_{\theta_x,{\theta}_y}^R\left({\mu}_x,{\mu}_y\right)=\exp \left[-\frac{{\left|I\left({\mu}_x,{\mu}_y\right)-I\Big({\theta}_x,{\theta}_y\Big)\right|}^2}{2{\sigma}_R^2}\right] $$

and

4 $$ {W}_{\theta_x,{\theta}_y}^M\left({\mu}_x,{\mu}_y\right)=\exp \left[-\frac{{\left|{I}^M\left({\mu}_x,{\mu}_y\right)-{I}^M\Big({\theta}_x,{\theta}_y\Big)\right|}^2}{2{\sigma}_M^2}\right] $$

where *I*(*μ*_*x*_, *μ*_*y*_)is the intensity value at(*μ*_*x*_, *μ*_*y*_) and *I*^*M*^(*μ*_*x*_, *μ*_*y*_) is the median filtered image at location (*μ*_*x*_, *μ*_*y*_). In addition to median filtering, the collateral filter introduces an entropy function that is utilized to balance the radiometric and median-filtered components using

5 $$ H\left({\theta}_x,{\theta}_y\right)=-{\sum}_{\left({\mu}_x,{\mu}_y\right)\in {\Psi}_{\theta_x,{\theta}_y}}p\left({\mu}_x,{\mu}_y\right)\log p\left({\mu}_x,{\mu}_y\right)+\left[1-p\left({\mu}_x,{\mu}_y\right)\right]\log \left[1-p\left({\mu}_x,{\mu}_y\right)\right] $$

where

6 $$ p\left({\mu}_x,{\mu}_y\right)=\frac{{\left[1-\frac{\left|I\left({\mu}_x,{\mu}_y\right)-{I}^M\Big({\mu}_x,{\mu}_y\Big)\right|}{{\mathit{\operatorname{MAX}}}_I}\right]}^2}{2}+0.5 $$

where *MAX*_*I*_ is the maximum intensity value of the input image *I*.

The ensemble weight function that combines all the components is then defined as

7 $$ {W}_{\theta_x,{\theta}_y}={W}_{\theta_x,{\theta}_y}^S{\left({W}_{\theta_x,{\theta}_y}^R\right)}^{\frac{1}{1+H\left({\theta}_x,{\theta}_y\right)}}{\left({W}_{\theta_x,{\theta}_y}^M\right)}^{H\left({\theta}_x,{\theta}_y\right)} $$

The collateral filtering of image *I* at location(*θ*_*x*_, *θ*_*y*_) is finally computed using

8 $$ {I}^C\left({\theta}_x,{\theta}_y\right)=\frac{\sum_{\left({\mu}_x,{\mu}_y\right)\in \Psi}{W}_{\theta_x,{\theta}_y}\left({\mu}_x,{\mu}_y\right)\left[\left(1-\beta \right)I+\beta {I}^M\right]}{\sum_{\left({\mu}_x,{\mu}_y\right)\in \Psi}{W}_{\theta_x,{\theta}_y}\left({\mu}_x,{\mu}_y\right)} $$

where *β* is adopted to adjust the weight between the input image *I* and the median filtered image *I*^*M*^. A number of techniques have been proposed to accelerate image filter computation. Some researchers used approximation methods to reduce the computation time [23]. A straightforward approach is to take advantage of an additional memory space. Inspired by the strategy in [24], we introduce a new scheme not only saving computation time but also achieving the same restoration results as using the traditional collateral filter.

In the traditional collateral filter, the spatial component is repeatedly computed for every pixel, which takes a lot of time in computing the same pixel distances. Therefore, we redefine (2) as

9 $$ DS\left({d}_x,{d}_y\right)=\exp \left[-\frac{{d_x}^2+{d_y}^2}{2{\sigma}_S^2}\right] $$

where *d*_*x*_ and *d*_*y*_ are the spatial distances with 0 ≤ *d*_*x*_ ≤ *N* and 0 ≤ *d*_*y*_ ≤ *N*. In our approach, we create a spatial weight buffer *WSB*, which is defined as

10 $$ WSB=\left\{ DS\left({d}_x,{d}_y\right):{d}_x,{d}_y\in \left[1,\dots, N\right]\right\} $$

We then compute all the spatial weights and store them in this memory buffer *WSB* according to the filter radius *N*. Let *WSB*(*x*, *y*) be an element of *WSB*, (2) can be expressed as *WSB*(*μ*_*x*_ − *θ*_*x*_, *μ*_*y*_ − *θ*_*y*_).

Subsequently, we implement the complex parts of the collateral filter using CUDA shared memory, which is an efficient way to reduce the reading and writing time comparing to the usage of global memory. We split the image pixels into separate blocks, each denoted as block(*i*, *j*), where *i* = 1, 2, …, *k* and *j* = 1, 2, …, *k*. For entropy computation, each pixel and its neighbors are required to sum up as shown in (5). To hasten the computation, the entropy of the pixel at (x, y) is computed and stored in the shared memory buffer in advance using

11 $$ {HPB}_{i,j}\left(x,y\right)=p\left(x,y\right)\log p\left(x,y\right)+\left[1-p\left(x,y\right)\right]\log \left[1-p\left(x,y\right)\right] $$

where *S* × (*i* − 1) − *N* < *x* ≤ *S* × *i* + *N*, *S* × (*j* − 1) − *N* < *y* ≤ *S* × *j* + *N*, and *S* × *S* represents the block size. Once *HPB*_*i*, *j*_ is created, the entropy is defined as:

12 $$ {HG}_{i,j}\left({\delta}_x,{\delta}_y\right)=-{\sum}_{\left({\mu}_x,{\mu}_y\right)\in {\Psi}_{\delta_x,{\delta}_y}}{HPB}_{i,j}\left({\mu}_x,{\mu}_y\right) $$

where (*δ*_*x*_, *δ*_*y*_) represents a thread in block(*i*, *j*) with 1 ≤ *δ*_*x*_ ≤ *S* and 1 ≤ *δ*_*y*_ ≤ *S*. Since accessing shared memory is faster than accessing local memory, each thread only needs to sum up its own value from its neighbors. Consequently, we redefine (7) as:

13 $$ {W}_{i,j,{\delta}_x,{\delta}_y}={WSB}_{i,j}{\left({W}_{i,j,{\delta}_x,{\delta}_y}^R\right)}^{\frac{1}{1+{HG}_{i,j}\left({\delta}_x,{\delta}_y\right)}}{\left({W}_{i,j,{\delta}_x,{\delta}_y}^M\right)}^{HG_{i,j}\left({\delta}_x,{\delta}_y\right)} $$

where *WSB*_*i*, *j*_ represents the spatial weight buffer in block(*i*, *j*).

**Fig. 1:**
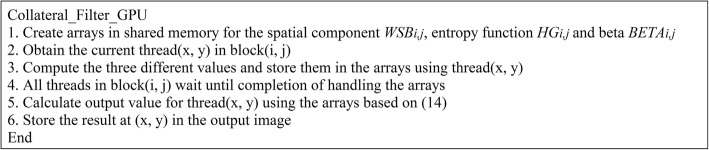
Pseudo code of the GPU-accelerated collateral filter

In order to more effectively utilize shared memory, the computation of *β* in (8) is reformulated so that it is calculated at the beginning in every block(*i*, *j*) before applying the filter to each pixel. Finally, the accelerated version of the collateral filter is defined as:

14 $$ {I}_{i,j}^{AC}\left({\delta}_x,{\delta}_y\right)=\frac{\sum_{\left({\mu}_x,{\mu}_y\right)\in {\Psi}_{\delta_x,{\delta}_y}}{W}_{i,j,{\delta}_x,{\delta}_y}\left({\mu}_x,{\mu}_y\right){BETA}_{i,j}\left({\mu}_x,{\mu}_y\right)}{\sum_{\left({\mu}_x,{\mu}_y\right)\in {\Psi}_{\delta_x,{\delta}_y}}{W}_{i,j,{\delta}_x,{\delta}_y}\left({\mu}_x,{\mu}_y\right)} $$

where *BETA*_*i*, *j*_(*μ*_*x*_, *μ*_*y*_) is the value of *β* at (*μ*_*x*_, *μ*_*y*_). The pseudo code of our GPU-based collateral filter is depicted in Fig. 1. Note that each pixel being processed is assigned to a thread since the process is a slice-by-slice approach, i.e., one 2D image at a time.

### Feature extraction

Although the execution time of the traditional collateral filter is hastened using the GPU strategy, it is still laborious to manually adjust the parameters for achieving the best restoration results in a particular brain MR image. Using an artificial neural network system is suitable as it can learn to estimate the optimal parameters in the filtering process. However, neural networks require appropriate input features to obtain the best performance of the predictable model. In our experience, image texture features play an important role in the neural network automation process. The candidate image texture features adopted in this study are described as follows.

#### Statistical features

There are several basic statistical features that are computed directly from the image intensity: mean intensity (Mean), standard deviation (SD), variance (VAR), entropy (ENT), and histogram features (skewness, kurtosis, variance, entropy and energy).

#### Gray-level co-occurrence matrix (GLCM)

The GLCM features were proposed by Haralick et al. [25]. A co-occurrence matrix is defined as a matrix of frequencies at which two pixels, separated by a certain distance and direction, occur in the image. Eight GLCM features [25–27] in four directions (*θ =* 0°, 45°, 90°, 135°) with distance *d =* 1 are computed:

1. Standard deviations in the *i-*direction (SD_*i*_)
2. Standard deviations in the *j-*direction (SD_*j*_)
3. Energy or angular second moment (ASM)
4. Contrast (CON)
5. Dissimilarity (DIS)
6. Homogeneity (HOM)
7. Entropy (ENT)
8. Correlation (COR)

#### Gray-level run-length matrix (GLRLM)

The GLRLM features were proposed by Galloway [28]. A run-length matrix is defined as the number of runs with a pixel of a specific gray level and its run length in a certain direction. Eleven GLRLM features in four directions (θ = 0°, 45°, 90°, 135°) are computed:

1. Short run emphasis (SRE)
2. Long run emphasis (LRE)
3. Gray level nonuniformity (GLN)
4. Run length nonuniformity (RLN)
5. Run percentage (RP)
6. Low gray-level run emphasis (LGRE)
7. High gray-level run emphasis (HGRE)
8. Short run low gray-level emphasis (SRLGE)
9. Short run high gray-level emphasis (SRHGE)
10. Long run low gray-level emphasis (LRLGE)
11. Long run high gray-level emphasis (LRHGE)

#### Tamura texture features

Tamura features [29] were developed based on the human visual and psychological perception. The first three features, which are correlated more closely with human perception, are computed:

1. Coarseness (CRS)
2. Contrast (CON)
3. Directionality (DIR)

Their formulas are briefly provided in Additional file 1.

#### Noise estimation features

Since the collateral filter parameters are sensitive to noise, noise related features are required. Many noise variance estimation methods that are based on the Rician and Rayleigh distributions are complicated. An easier way to estimate the noise level is to employ a simpler Laplacian operator [30]. A more efficient algorithm [31] with a generic transfer function improved the Laplacian mask for better estimation. Aja-Fernández et al. [32] presented a set of estimators based on the Rayleigh distribution in the background. Moreover, the peak value of the autocorrelation function computed by an image with itself provides the information of noise [33]. Based on the above methods, the following seven features are adopted:

1. Laplacian mask (LAP)
2. Laplacian of Gaussian (LOG)
3. A generic transfer function with Laplacian (GTFLAP)
4. Maximum value of some local distribution (AJA)
5. A least square fitting of the Rayleigh distribution with the histogram background (BRUM)
6. Maximum likelihood function (SIJ)
7. Autocorrelation function (ACF)

### Feature selection

If a large amount of features are selected as the input arguments, the training of neural networks will be an extremely time-consuming procedure. Consequently, we utilize the sequential forward floating selection (SFFS) method [34] to choose the best combination of features. Before applying the SFFS, we make use of the paired-samples t-test [35, 36] to rank the significance of candidate features. The t-test is applied to each image feature to evaluate the ability for discriminating differences between noise levels, which results in an average *p*-value. The smaller the *p*-value, the better the discrimination. Statistically, those features with an average *p* < 0.05 are selected as candidates for the SFFS process.

### Neural networks

The popular back propagation neural network (BPN), proposed by McClelland et al. [37], is utilized for training our neural network system. The main architecture includes input, hidden and output layers. The back propagation process is employed to optimize the weights by minimizing the target error so that the neural network is able to learn the input-output mapping perfectly. In our approach, the inputs are the features selected by the SFFS algorithm and the outputs are the collateral filter parameters. In addition, a brute-force approach is conducted to obtain the optimal filter parameters based on the peak signal-to-noise ratio (PSNR):

15 $$ PSNR=10\times {\log}_{10}\left[\frac{{\mathit{\operatorname{MAX}}}_I^2}{\frac{1}{mn}\times \sum \limits_{i=0}^{m-1}\sum \limits_{j=0}^{n-1}{\left[R\left(i,j\right)-F\Big(i,j\Big)\right]}^2}\right] $$

where *F* is the *m* × *n* noise-free image, *R* is the restored image, and *MAX*_*I*_ is the maximum possible intensity of *F*. The optimal filter parameters are decided when the highest *PSNR* value is obtained.

**Fig. 2:**
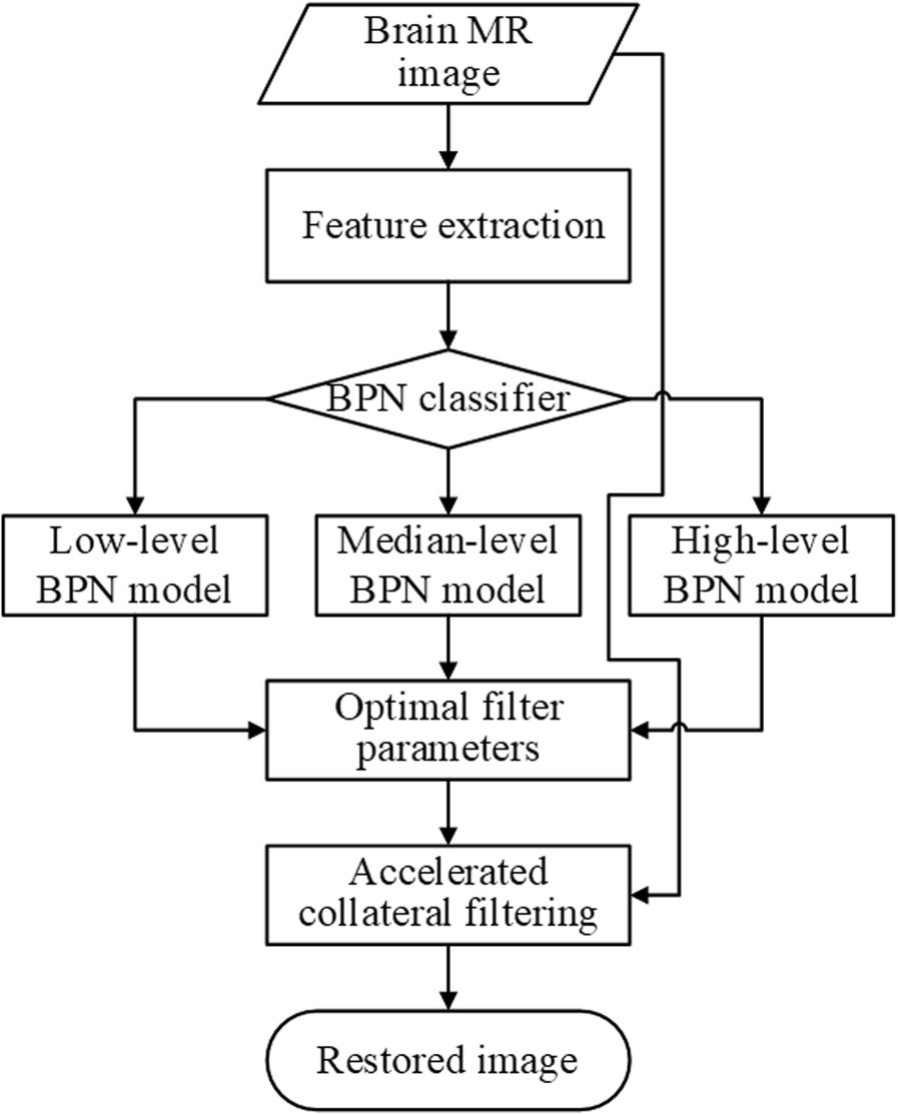
Flowchart of the proposed restoration framework with automatic parameter decision

Finally, a two stage neural network structure is developed with the first stage for classification and the second stage for prediction. At the first stage, the neural network classifier distinguishes inputs into three different noise levels. Multiple-layer (input, hidden, and output) neural networks with different noise level models at the second stage predict the optimal filter parameters for image restoration. The Levenberg-Marquardt learning algorithm [38] is exploited to independently train each of the three individual BPN models. For each BPN model, the transfer function between the input layer and the hidden layer is the hyperbolic tangent function, whereas the linear transfer function is employed between the hidden layer and the output layer. The overall flowchart of the proposed methods is depicted in Fig. 2.

### Performance evaluation

In addition to the PSNR metric in (15), the structural similarity (SSIM) metric [39] is utilized to evaluate the restored images using

16 $$ \mathrm{SSIM}\left(F,R\right)=\frac{\left(2{\mu}_F{\mu}_R+{C}_1\right)\left(2{\sigma}_{FR}+{C}_2\right)}{\left({\mu}_F^2+{\mu}_R^2+{C}_1\right)\left({\sigma}_F^2+{\sigma}_R^2+{C}_2\right)} $$

where *σ* represents the standard deviation, *μ* represents the mean intensity, *C*_1_ = (*K*_1_*MAX*_*I*_)^2^ and *C*_2_ = (*K*_2_*MAX*_*I*_)^2^ with *K*_1_ = 0.01 and *K*_2_ = 0.03. The higher the PSNR and SSIM scores the better the restoration results. To more thoroughly evaluate the image restoration ability of the proposed system, the restoration results obtained using the automatic framework are compared with the results obtained using the brute-force method, which is considered as the optimal outcome. Let PSNR_A_ be the restoration score of the proposed automatic system and PSNR_T_ be the restoration score of the brute-force method. The relative error *ε*_*r*_ is then defined as

17 $$ {\varepsilon}_r=\frac{\left|{\mathrm{PSNR}}_{\mathrm{T}}-{\mathrm{PSNR}}_{\mathrm{A}}\right|}{{\mathrm{PSNR}}_{\mathrm{T}}}\times 100\% $$

## Results

We first conducted the experiments on the BrainWeb database [40] as it contains simulated brain MR image data based on normal and multiple sclerosis (MS) models with different thicknesses, noise levels and intensity non-uniformities. Clinical image data were acquired from the medical image database in the Division of Interventional Neuro Radiology, Department of Radiology, UCLA, Los Angeles, CA, USA. For efficiency tests, the experiments were performed on an Intel® Xeon(R) CPU E5–2620 v3 2.40GHz equipped with a Tesla K40c GPU. Tesla K40c is one NVIDIA’s GPU based on the NVIDIA’s Kepler architecture. The Tesla K40c GPU contains 15 multiprocessors and each multiprocessor contains 192 cores, which results in 2880 cores. Its memory size is 12 GB and the maximum bandwidth is 288 (GB/sec). Each block has 65,536 registers with 48 KB shared memory and the maximum number of threads is 1024. The CUDA driver version is 7.5. The algorithm was implemented and programmed in MATLAB 2017a (The MathWorks Inc. Natick, MA, USA) associated with C for CUDA-based acceleration.

**Fig. 3:**
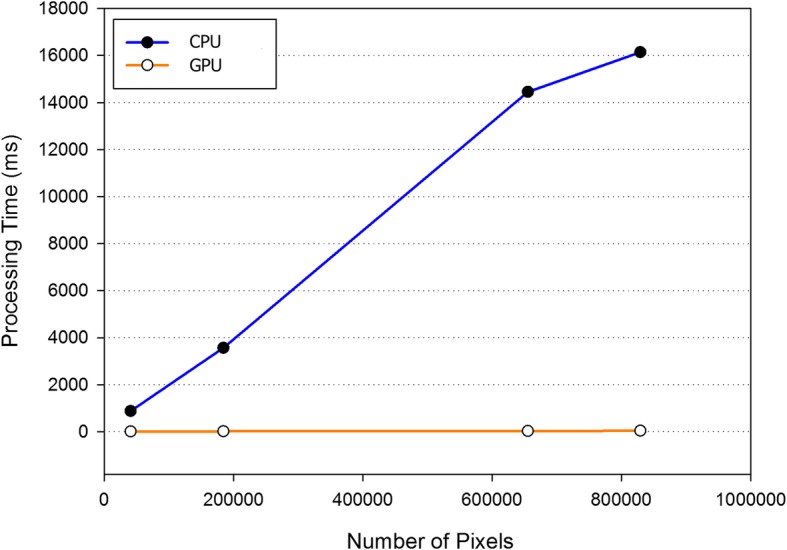
Plots of the running times of the CPU-based and the GPU-based with shared memory collateral filters

Table 1 presents the execution performance of the traditional collateral filter and our accelerated version on the same 217 × 181 brain MR images. The performance of using the shared memory (SM) or not is also indicated. The block size for the shared memory was 16 × 16 threads and the filter size was 3 × 3. When the number of images was increasing, the proposed framework executed the filtering process more effectively and a remarkable acceleration was attained. It was noted that the speed up gain raised from 34 for one single image to 541 for processing 100 images. Our proposed GPU-based collateral filter is much faster than the original CPU version. The corresponding running times with respect to different image pixel sizes are depicted in Fig. 3. Obviously, the GPU implementation ran less than 0.1 s for each scenario, whereas the computation time of the CPU version was roughly linearly proportional to the pixel number.

**Table 1:** Execution time of the CPU-based and GPU-based collateral filters

Image number	CPU (s)	GPU without SM (s)	GPU with SM (s)	Speed up
1	0.63	0.14	0.018	34
10	6.06	0.16	0.027	224
100	58.78	0.24	0.108	541
500	260.52	0.59	0.425	612
1000	512.38	1.05	0.871	588

As presented in Table 2, there were 28 selected features from different image feature classes for the input candidates of the BPN system. The order of significance of these adopted image features was based on the average *p*-value of the t-test outcome with *p* < 0.05. For understanding the optimal features, T1-weighted brain MR images with three different slice thicknesses (1 mm, 3 mm and 5 mm), five noise levels (1, 3, 5, 7 and 9%), and three intensity non-uniformities (0, 20 and 40%) acquired from the BrainWeb database were utilized to evaluate the proposed automatic restoration framework. Each noise level had 1476 images, which were divided into two groups: 984 slices for training and 492 slices for testing. After the SFFS procedure, five optimal features of RLN(0°), RP(135°), RP(90°), AJA, and kurtosis were obtained and they were further employed in the BPN training phase.

**Table 2:** Paired-samples t-test results based on the average *p*-value with *p* < 0.05

No.	Feature	*p*-value	No.	Feature	*p*-value
1	BRUM	0.019017	15	AJA	0.038044
2	SIJ	0.020022	16	GLN(45°)	0.038485
3	SRLGE(90°)	0.021380	17	RP(45°)	0.040108
4	GTFLAP	0.022898	18	RP(135°)	0.040233
5	SRLGE(45°)	0.023014	19	LGRE(0°)	0.041105
6	SRLGE(0°)	0.028114	20	LGRE(90°)	0.041233
7	SRLGE(135°)	0.028445	21	RLN(135°)	0.042278
8	RLN(90°)	0.030137	22	SIJ	0.043803
9	GLN(0°)	0.032553	23	LAP	0.044592
10	RP(0°)	0.032900	24	LGRE(135°)	0.045512
11	RP(90°)	0.033309	25	GLN(135°)	0.046477
12	GLN(90°)	0.033748	26	RLN(45°)	0.046907
13	RLN(0°)	0.034005	27	ACF	0.048627
14	con	0.035178	28	LOG	0.049145

In the testing phase, the first stage BPN system classified the input image into three major categories based on the noise level: low (1 and 3%), median (5 and 7%) and high (9%) estimators. The correct classification rate was up to 97.6% overall. The computed five features of the input image were fed into the corresponding second stage BPN model for producing the filter parameter values for denoising. Table 3 summarizes the PSNR scores and the relative error *ε*_*r*_ of the proposed automatic filtering framework and the brute-force method on randomly selected slices of the 5 mm normal MR images with 5 and 7% noise. It is indicated that the PSNR scores of the proposed restoration algorithm were extremely close to the optimal PSNR scores of the brute-force method with negligible errors.

**Table 3:** Restoration result analyses of the proposed GPU-based collateral filter on different slices

Slice		7	14	17	20	23
Noise						
5%	PSNR_A_	33.066	34.131	34.156	33.836	34.736
	PSNR_T_	33.229	34.145	34.157	33.855	34.758
	*ε*_*r*_(%)	0.4891	0.0395	0.0031	0.0575	0.0621
7%	PSNR_A_	31.278	31.728	31.956	32.015	32.613
	PSNR_T_	31.355	31.837	31.960	32.028	32.658
	*ε*_*r*_(%)	0.2453	0.3430	0.0117	0.0415	0.1377

**Fig. 4:**
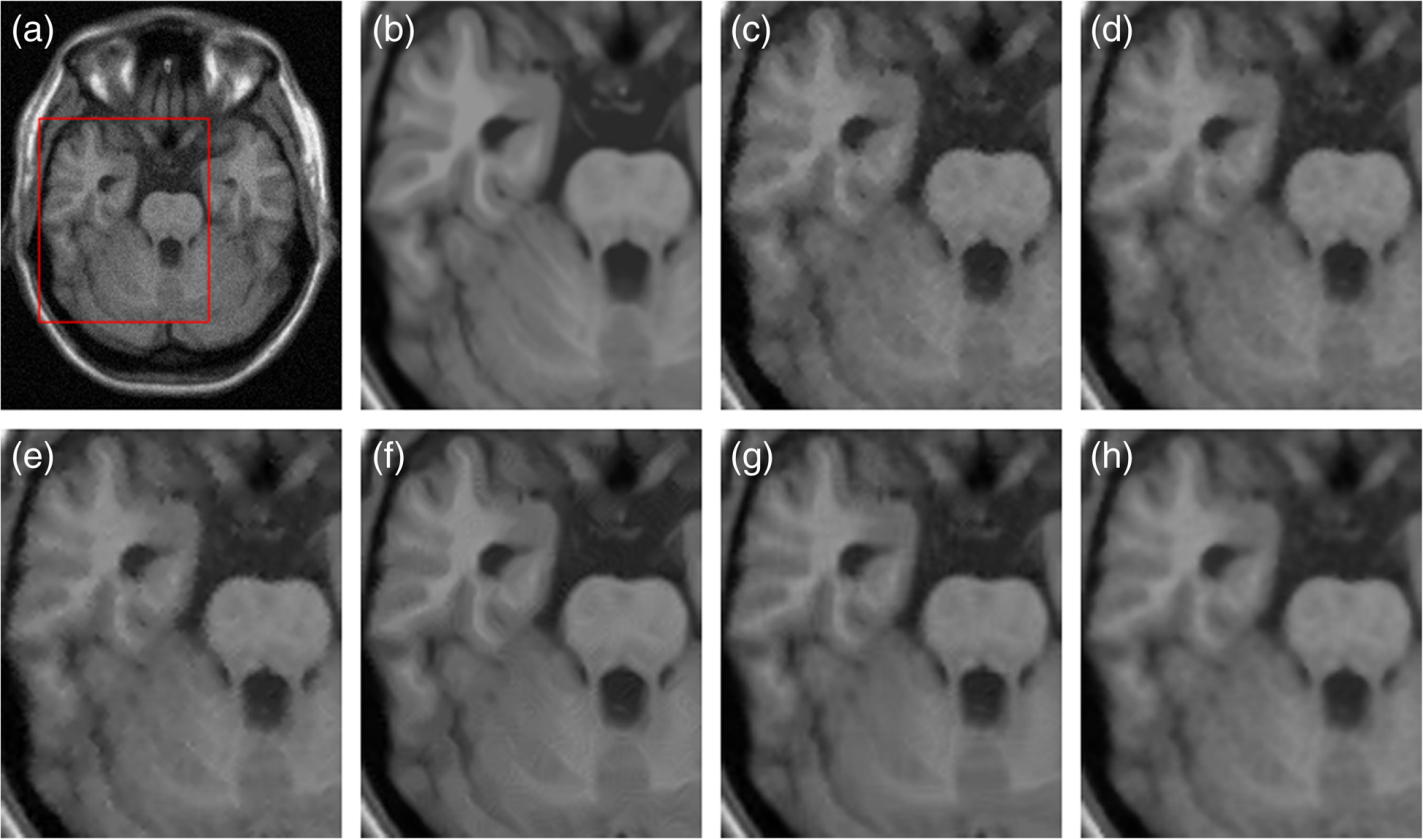
Comparison of visual restoration results on slice 10 with 5% noise in the 5 mm MS dataset. **a** Noisy MR image. **b** Intact image in magnified view. **c** IBF with PSNR = 32.49 and SSIM = 0.8650. **d** ADF with PSNR = 33.61 and SSIM = 0.8684. **e** LMMSE with PSNR = 32.53 and SSIM = 0.8657. **f** NLM with PSNR = 32.53 and SSIM = 0.8800. **g** BM3D with PSNR = 32.49 and SSIM = 0.8997. **h** Proposed with PSNR = 34.32 and SSIM = 0.8803

**Fig. 5:**
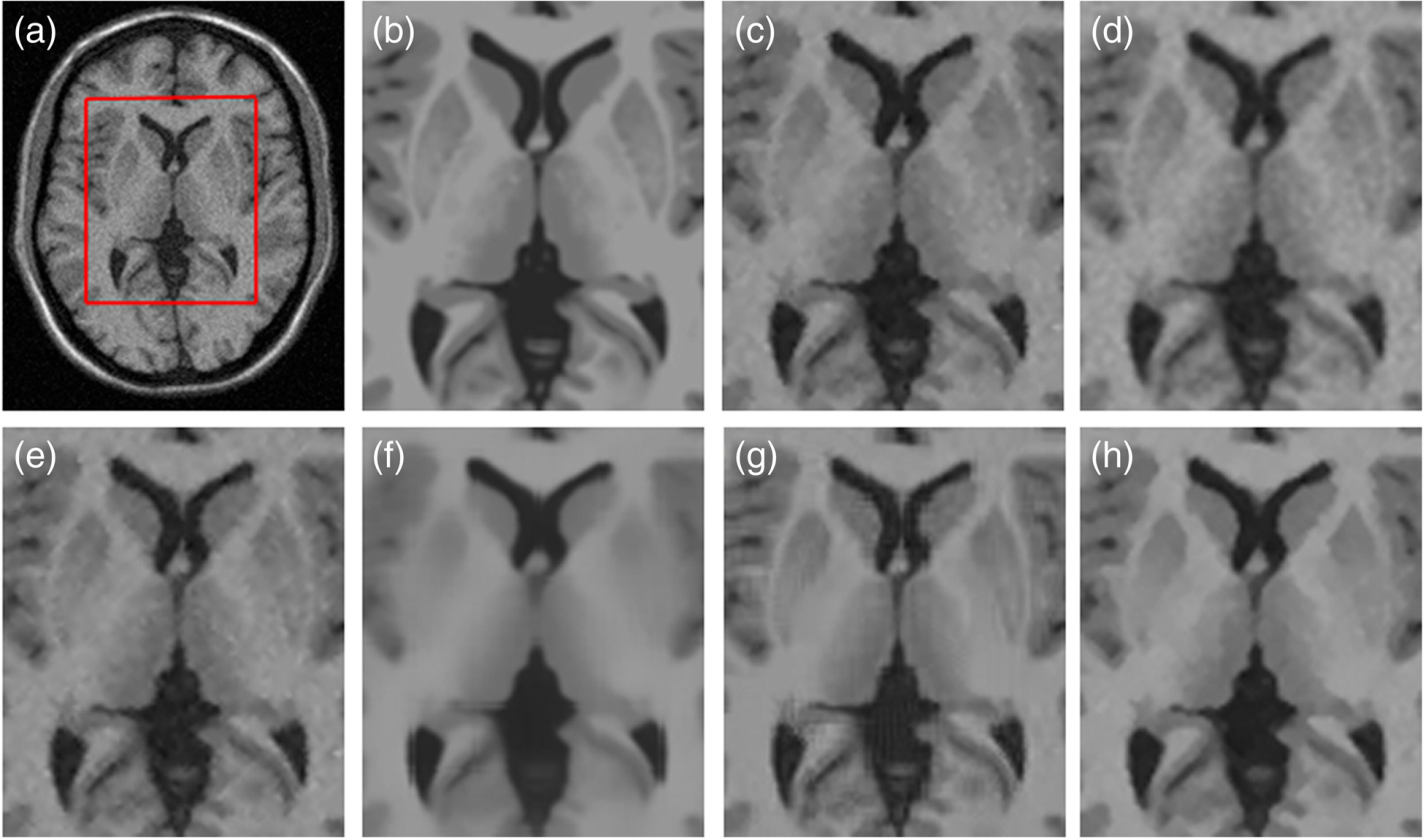
Comparison of visual restoration results on slice 26 with 7% noise in the 3 mm MS dataset. **a** Noisy MR image. **b** Intact image in magnified view. **c** IBF with PSNR = 28.87 and SSIM = 0.7981. **d** ADF with PSNR = 31.41 and SSIM = 0.8133. **e** LMMSE with PSNR = 29.08 and SSIM = 0.8016. **f** NLM with PSNR = 31.74 and SSIM = 0.8406. **g** BM3D with PSNR = 31.78 and SSIM = 0.8367. **h** Proposed with PSNR = 31.95 and SSIM = 0.8479

In Fig. 4, we illustrate our automatic restoration outcome of the 10th slice image corrupted by 5% noise level in the 5 mm MS dataset in comparison with the ADF [9], LMMSE [8], NLM [12], iterative bilateral filtering (IBF) [7], and BM3D [13] methods. Comparing to other techniques, the proposed GPU-based collateral filter revealed more uniform intensity profiles and better sharpened edges in the white matter (WM) and gray matter (GM) regions. Quantitative analyses also indicated that our automatic collateral filter provided the highest score of PSNR = 34.32 dB and the second highest SSIM = 0.8803 than other tested methods. Figure 5 depicts the visual restoration results of the 26th slice image corrupted by 7% noise level in the 3 mm MS dataset. It was obvious that, in contrast to other methods, the proposed denoising algorithm achieved a sharper and cleaner representation for the GM and WM with the best PSNR = 31.95 dB and SSIM = 0.8479, which was closer to the intact image.

**Fig. 6:**
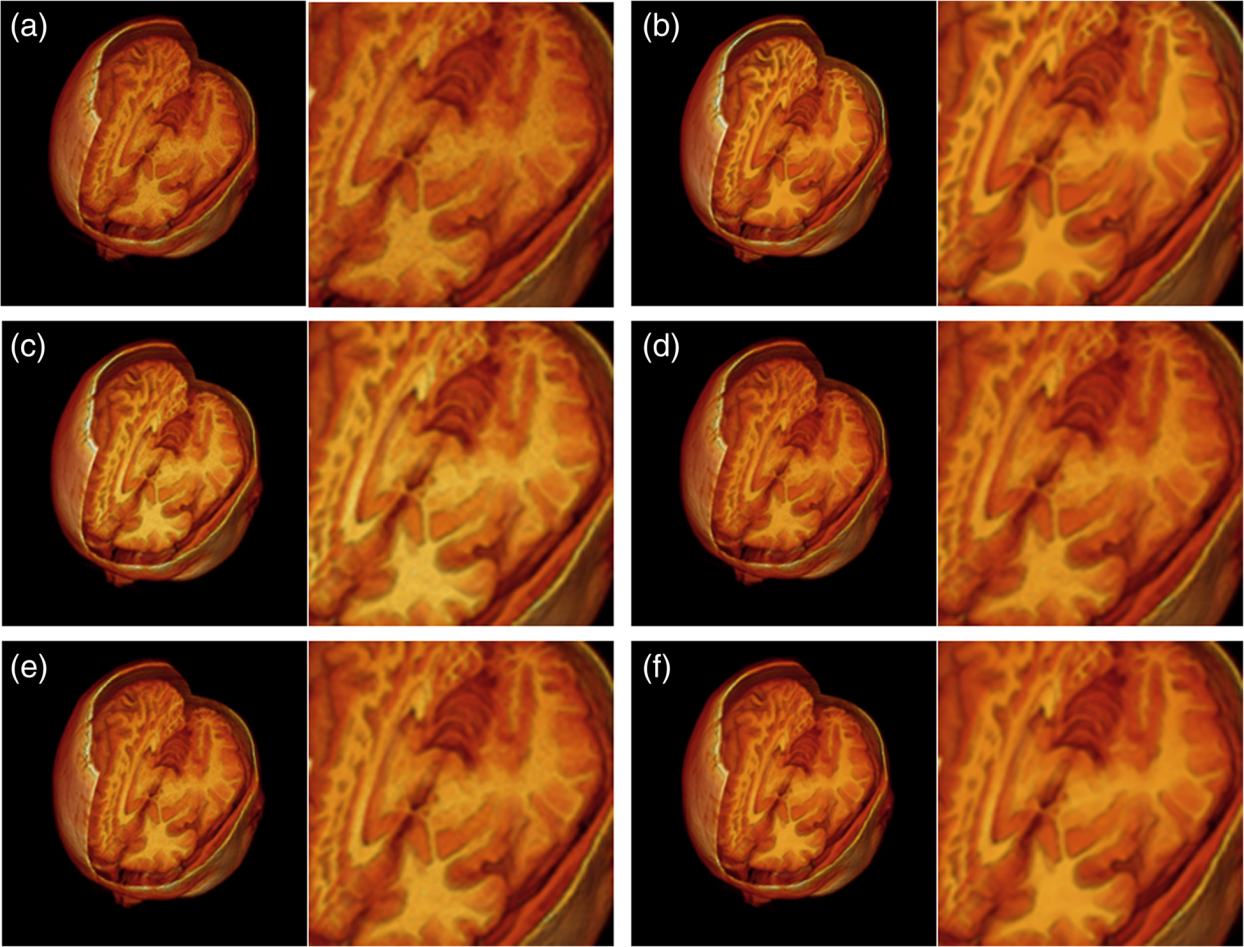
Qualitative analyses on the restoration results of the 1 mm normal image dataset with 9% noise and 40% intensity non-uniformity in 3D visualization. **a** Noisy image volume. **b** Intact image volume. **c** ADF. **d** LMMSE. **e** IBF. **f** Proposed

Figure 6 delineates the restoration results of the 1 mm normal image volume with 9% noise level and 40% intensity non-uniformity in 3D visualization. After applying the ADF, LMMSE, and IBF methods, the brain structures still, more or less, exhibited grainy noise influences. On the other hand, the proposed automatic filter decently wiped out the heavy noise while maintaining noticeable cortical structures as illustrated in Fig. 6f. Table 4 presents quantitative analyses on massive 1 mm image volume restoration with various scenarios of anatomical models and noise levels. It was noted that the LMMSE method performed much worse than other methods in the image volumes with 1% noise. Our automatic denoising framework produced the best restoration results in terms of average PSNR and SSIM in all noise levels. As the noise level was increasing, the advantage of our collateral filter over other methods was more compelling.

**Table 4:** Quantitative analyses on the restoration results of the 1 mm image data in terms of average PSNR and SSIM scores using different methods

Type	Noise	LMMSE		IBF		Proposed	
		PSNR	SSIM	PSNR	SSIM	PSNR	SSIM
Normal	1%	34.97	0.8513	38.04	0.9766	39.17	0.9816
	3%	33.34	0.8626	33.28	0.8924	34.79	0.9027
	5%	30.65	0.8042	30.51	0.8239	32.42	0.8538
	7%	28.65	0.7535	28.60	0.7711	30.53	0.7959
	9%	27.03	0.7127	27.04	0.7309	29.13	0.7585
MS	1%	35.09	0.8512	38.16	0.9767	39.29	0.9830
	3%	33.48	0.8628	33.40	0.8928	34.87	0.9006
	5%	30.79	0.8046	30.64	0.8244	32.51	0.8540
	7%	28.79	0.7541	28.73	0.7715	30.62	0.7958
	9%	27.17	0.7135	27.16	0.7315	29.21	0.7587

**Fig. 7:**
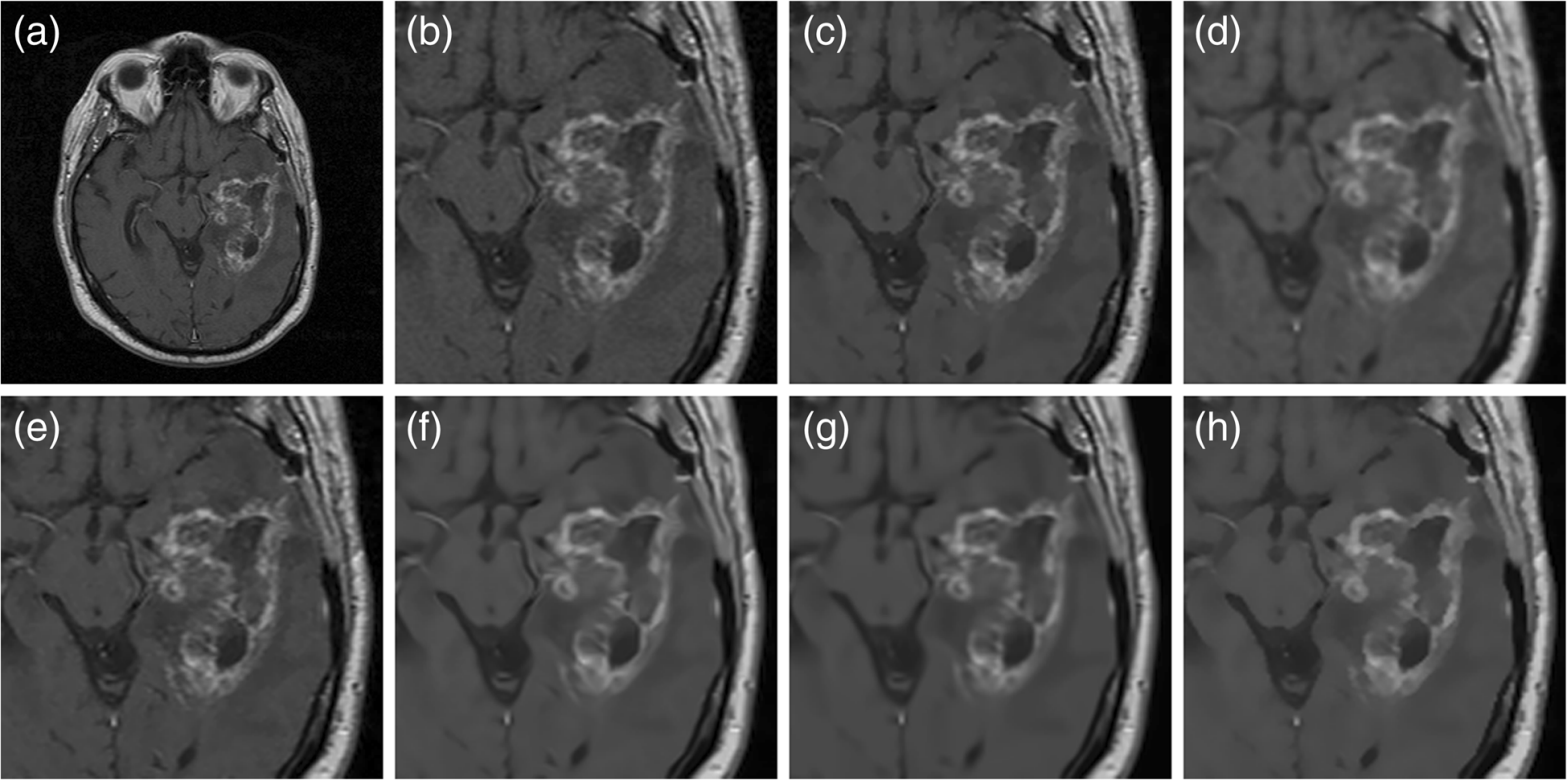
Comparison of the restoration of clinical T1-weighted MR images with brain tumors. **a** Input MR image. **b** Input image in magnified view. **c** IBF. **d** ADF. **e** LMMSE. **f** NLM. **g** BM3D. **h** Proposed

**Fig. 8:**
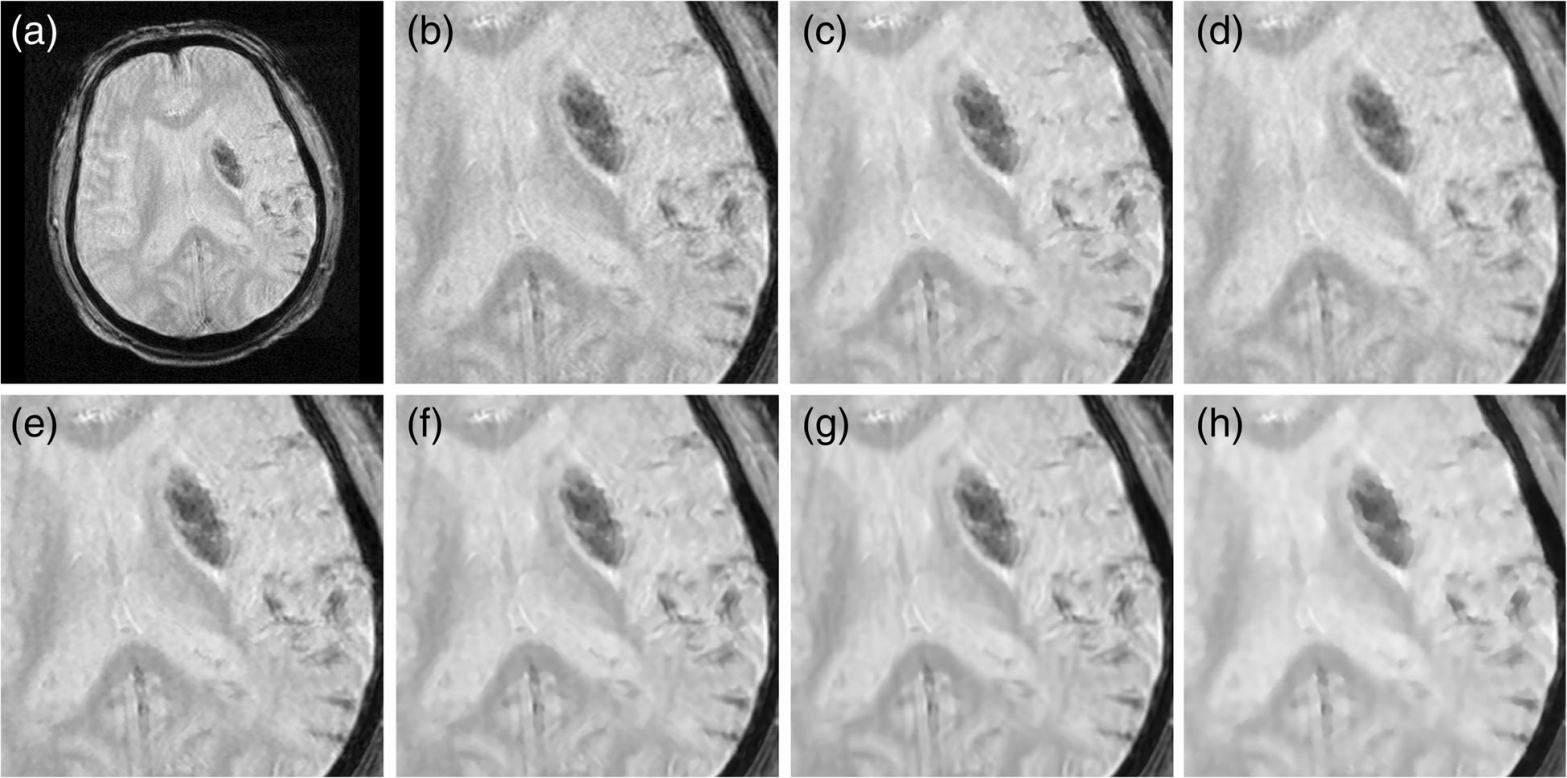
Comparison of the restoration of distorted PD-weighted MR images with brain tumors. **a** Input MR image. **b** Input image in magnified view. **c** IBF. **d** ADF. **e** LMMSE. **f** NLM. **g** BM3D. **h** Proposed

**Fig. 9:**
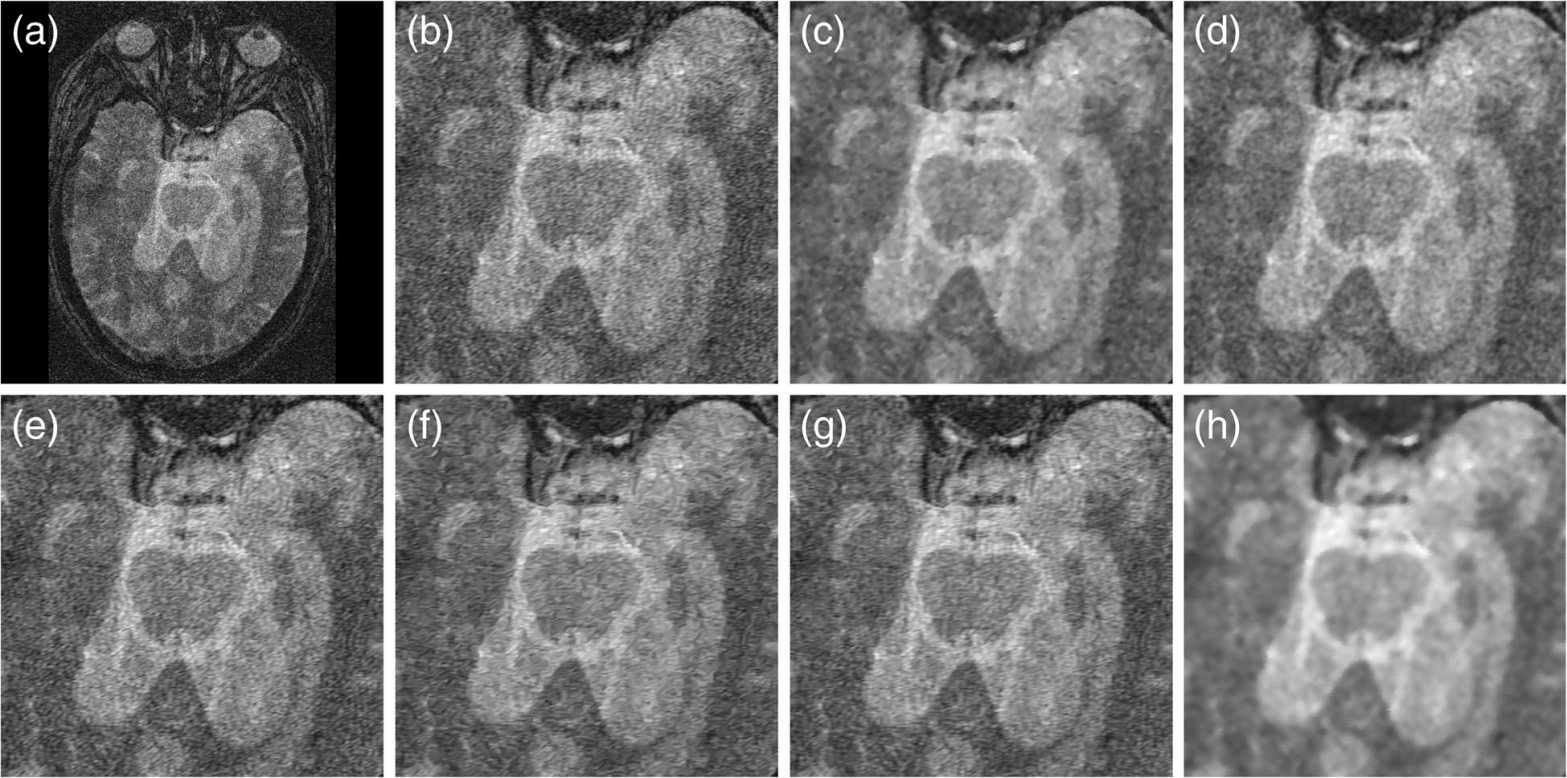
Comparison of the restoration of noisy MR images with low resolution. **a** Input MR image. **b** Input image in magnified view. **c** IBF. **d** ADF. **e** LMMSE. **f** NLM. **g** BM3D. **h** Proposed

For completeness, we demonstrate the capability of our automatic filtering algorithm in restoring clinical brain MR images with diseases. Figure 7 depicts the restoration results of denoising T1-weighted MR images with brain tumors using different methods. While the restoration results obtained using the ADF, LMMSE, and IBF methods were somewhat grainy in the tumor area, the NLM, BM3D and proposed algorithms achieved more suitable smoothness while maintaining sharpness at the tumor boundaries. Another example of filtering distorted PD-weighted MR images with brain tumors is illustrated in Fig. 8. It was indicated that, comparing to other methods, our automatic collateral filter more effectively removed the artefact while disclosing distinct edges between anatomical structures. Finally, in Fig. 9, we show the visual improvement of noisy MR images with low resolution and severe grains using all tested methods. It was obvious that the proposed filtering algorithm more adequately eliminated the noise and identified the lesion in contrast to other techniques.

## Discussion

Stimulated by the recent advance in artificial intelligence and parallel computing, we have developed an efficient filtering framework for brain MR images. An essential property of the proposed automatic restoration algorithm is the two-stage mechanism of the neural network concatenation. A benefit of this strategy is to divide the broad noise range into three narrower classes corresponding to three individual BPN models, where the prediction of the filter parameters can be further clarified. To correlate the image with the BPN models for automation, a wide variety of image texture features were investigated based on the SFFS strategy, from which five best texture features were obtained. Three features of RLN(0°), RP(135°), and RP(90°) belong to the GLRLM category, AJA is one of the noise estimation features, and kurtosis makes use of the histogram information. These five features play an essential role in the effective discrimination between images with different noise levels, anatomical structures, and intensity non-uniformities.

A variety of T1-weighted brain MR images acquired from the BrainWeb database [40] with various scenarios were utilized in the proposed BPN models to obtain the optimal filter parameters. Due to the facility limitations, other MR modalities such as T2-weighted and PD-weighted images were not included in the training dataset in the current study. From the viewpoint of the clinical demand, the proposed automatic filter adequately restored various brain MR images with distinct anatomical structures and modalities such as PD-weighted images (see Figs. 7, 8, 9), which were quite different from the training BrainWeb dataset. This suggested the propriety of the proposed automation strategy and the robustness of the developed restoration scheme. Nevertheless, to achieve optimal restoration results on massive T2-weighted and PD-weighted brain images, retraining involving these types of images is recommended.

## Conclusions

We have described an automatic image restoration algorithm based on the accelerated collateral filter that has been implemented on the GPU architecture. The proposed framework reduced the execution time of the filtering process by taking advantage of shared memory in CUDA to decrease memory access latency. This new denoising system fully automated the restoration procedure for brain MR images without the need of adjusting the filter parameters. Experimental results indicated that the proposed automatic restoration framework effectively removed noise in various brain MR images with satisfactory quantity and quality. We also demonstrated its great capability of improving the clinical MR image quality and delineating the tumor boundary that outperformed other tested methods. We believe that our automatic filtering framework is of potential and able to provide a fast and powerful solution in a wide variety of medical image restoration applications.

## Additional file

Additional file 1: Formulae. (DOCX 19 kb)

## Abbreviations

ADF: Anisotropic diffusion filter
BPN: Propagation neural network
CPU: Central processing unit
CUDA: Compute unified device architecture
GLCM: Gray-level co-occurrence matrix
GLN: Gray level nonuniformity
GLRLM: Gray-level run-length matrix
GM: Gray matter
GPGPU: General purpose computation on GPU
GPU: Graphics processing unit
HGRE: High gray-level run emphasis
IBF: Iterative bilateral filtering
LGRE: Low gray-level run emphasis
LMMSE: Linear minimum mean square error
LOG: Laplacian of Gaussian
LRE: Long run emphasis
LRHGE: Long run high gray-level emphasis
LRLGE: Long run low gray-level emphasis
MRI: Magnetic resonance imaging
MS: Multiple sclerosis
PSNR: Peak signal-to-noise ratio
RLN: Run length nonuniformity
RP: Run percentage
SD: Standard deviation
SFFS: Sequential forward floating selection
SM: Shared memory
SRE: Short run emphasis
SRHGE: Short run high gray-level emphasis
SRLGE: Short run low gray-level emphasis
SSIM: Structural similarity
WM: White matter
