# Supplementary material for: An automatic restoration framework based on GPU-accelerated collateral filtering in brain MR images
Source: BMC Med Imaging. 2019 Jan 19;19:8. doi: 10.1186/s12880-019-0305-9 (PMC6339330; doi:10.1186/s12880-019-0305-9)
Supplement: Supplementary file 1 — Formulae. (DOCX 19 kb) [file 12880_2019_305_MOESM1_ESM.docx]

# Appendix

The three Tamura features, CRS, CON, and DIR, are computed as follows:

1. CRS

First, compute the average intensity value at every pixel $\left( x,y \right)$ over its neighborhoods whose sizes are integer powers of 2:

$A_{k}\left( x,y \right)=\frac{1}{2^{2k}}\sum_{i=x-2^{k-1}}^{x+2^{k-1}-1} \sum_{j=y-2^{k-1}}^{y+2^{k-1}-1} I\left( i,j \right)$ (A1)

where $I\left( i,j \right)$ is the gray level at $\left( i,j \right)$ and $k\in\left\langle0,\ldots,5 \right\rangle$. For each pixel $\left( x,y \right)$, calculate the differences between pairs of averages of non-overlapping neighborhoods on the opposite side of the point in both horizontal and vertical directions respectively as

$E_{k,h}\left( x,y \right)=\left| A_{k}\left( x+2^{k-1},y \right)-A_{k}\left( x-2^{k-1},y \right) \right|$ (A2)

$E_{k,v}\left( x,y \right)=\left| A_{k}\left( x,y+2^{k-1} \right)-A_{k}\left( x,y-2^{k-1} \right) \right|$ (A3)

Subsequently, select the best size that gives $S_{best}\left( x,y \right)=2^{k}$the highest output value, where $k$ maximizes $E$ in either direction:

$E_{k}\left( x,y \right)=\max_{} \left( E_{k,h}\left( x,y \right),E_{k,v}\left( x,y \right) \right)$ (A4)

Finally*,* $\mathrm{CRS}$ is measured by:

$\mathrm{CRS}=\frac{1}{m\times n}\sum_{i=1}^{m} \sum_{j=1}^{n} S_{best}\left( i,j \right)$ (A5)

where $m$ and $n$ are the effective width and height of the image, respectively.

1. CON

$CON=\frac{\sigma}{\left( \frac{\sum_{i=1}^{m} \sum_{j=1}^{n} {(I(i,j)-\mu)}^{4}}{m\times n\times\sigma^{4}} \right)^{t}}$ (A6)

where $\sigma$ is the variance of the gray-level probability distribution, $t$ is a positive number, and $\mu$ is the mean intensity.

1. DIR

$\mathrm{DIR}=1-r\times n_{p}\times\sum_{p}^{n_{p}} \sum_{\phi\in w_{p}} \left( \phi-\phi_{p} \right)^{2}H_{D}\left( \phi\right)$ (A7)

where $n_{p}$ is the number of peaks, $w_{p}$ is the range of the $p^{th}$ peak between valleys, $r$ is a normalizing factor related to quantizing levels of $\phi$, $\phi_{p}$ is the $p^{\mathrm{th}}$ peak position of $H_{D}$, and $H_{D}$ is the edge probability histogram by quantizing $\theta$ using

$\theta=\tan^{-1} \left( \frac{\Delta_{V}}{\Delta_{H}} \right)+\frac{\pi}{2}$ (A8)

and counting the points with the magnitude $\left| \Delta G \right|$ using

$\left| \Delta G \right|=\frac{\left| \Delta_{H} \right|+\left| \Delta_{V} \right|}{2}$ (A9)

with

$H_{D}\left( k \right)=\frac{N_{\theta}\left( k \right)}{\sum_{i=0}^{d-1} N_{\theta}\left( i \right)}, k=0, 1, \ldots, d-1$ (A10)

where $N_{\theta}\left( k \right)$ is the number of points at which ${\left( 2k-1 \right)\pi}/{2d\leq\theta<}{\left( 2k+1 \right)\pi}/{2d}$ and $\left| \Delta G \right|\geq\lambda$ with $\lambda$ a threshold. In (A8) and (A9), $\Delta_{V}$ and $\Delta_{H}$ represent the edge detection maps in the vertical and horizontal directions, respectively. The purpose of thresholding $\left| \Delta G \right|$ by $\lambda$ is to prevent counting unreliable directions that are not regarded as edge points.
